# Author Correction: Ribonucleotide incorporation into mitochondrial DNA drives inflammation

**DOI:** 10.1038/s41586-025-09682-9

**Published:** 2025-10-03

**Authors:** Amir Bahat, Dusanka Milenkovic, Eileen Cors, Mabel Barnett, Sadig Niftullayev, Athanasios Katsalifis, Marc Schwill, Petra Kirschner, Thomas MacVicar, Patrick Giavalisco, Louise Jenninger, Anders R. Clausen, Vincent Paupe, Julien Prudent, Nils-Göran Larsson, Manuel Rogg, Christoph Schell, Isabella Muylaert, Erik Larsson, Hendrik Nolte, Maria Falkenberg, Thomas Langer

**Affiliations:** 1 Max Planck Institute for Biology of Ageing, Cologne, Germany; 2 Institute for Biomedicine, University of Gothenburg, Gothenburg, Sweden; 3 Medical Research Council Mitochondrial Biology Unit, University of Cambridge, Cambridge, UK; 4 Department of Medical Biochemistry and Biophysics, Karolinska Institutet, Stockholm, Sweden; 5 Institute of Surgical Pathology, Faculty of Medicine, Medical Center—University of Freiburg, Freiburg, Germany; 6 Cologne Excellence Cluster on Cellular Stress Responses in Aging-Associated Diseases (CECAD), University of Cologne, Cologne, Germany; 7 Present Address: The CRUK Scotland Institute, Glasgow, UK

**Keywords:** Stress signalling, Mitochondria, Senescence

Correction to: *Nature* 10.1038/s41586-025-09541-7 Published online 24 September 2025

In the version of the article initially published, Erik Larsson’s surname was incorrect and has now been amended in the HTML and PDF versions of the article.

