# Supplementary material for: Ribonucleotide incorporation into mitochondrial DNA drives inflammation
Source: Nature. 2025 Sep 24;647(8090):726–34. doi: 10.1038/s41586-025-09541-7 (PMC12629987; doi:10.1038/s41586-025-09541-7)

nature portfolio

https://doi.org/10.1038/s41586-025-09541-7

Supplementary information

Ribonucleotide incorporation into mitochondrial DNA drives inflammation

In the format provided by the authors and unedited

Figure 4d

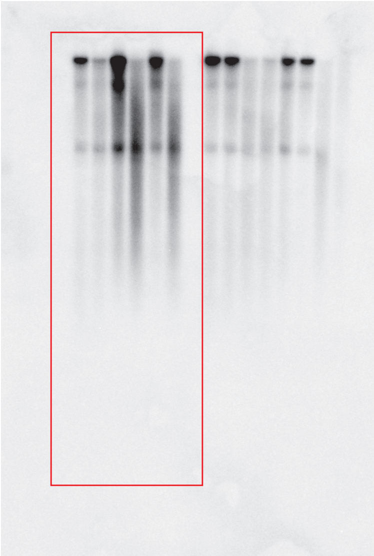

Figure 4f

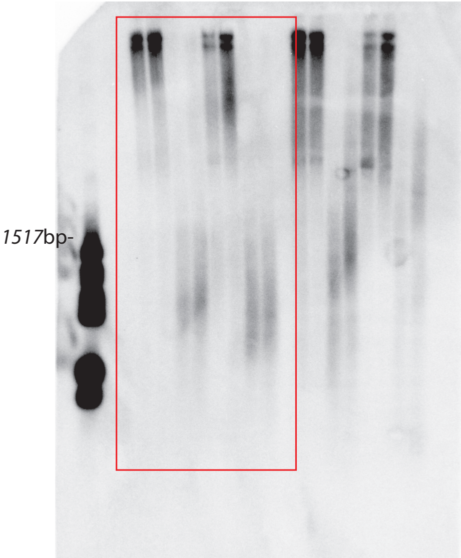

Figure 5b

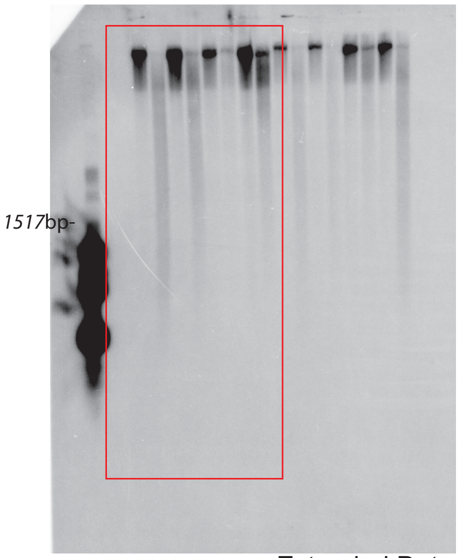

Figure5d

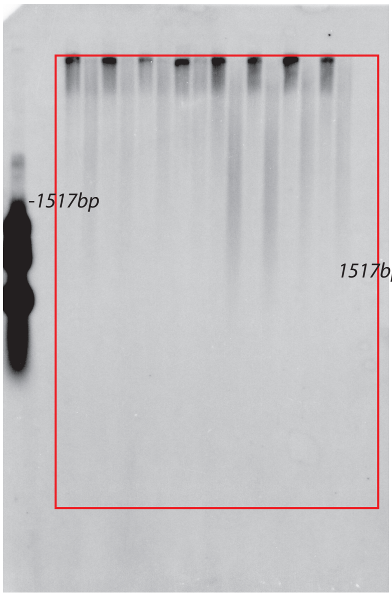

Figure 6b

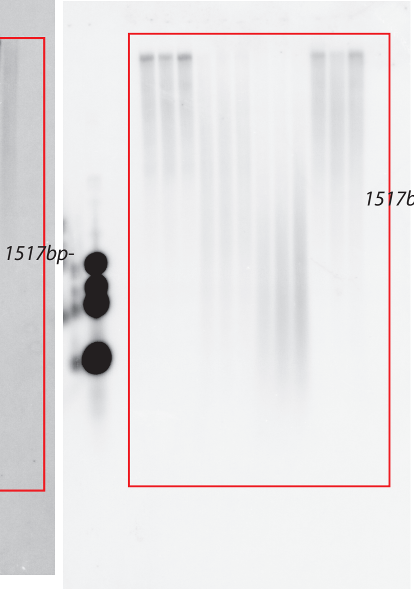

Figure 6d

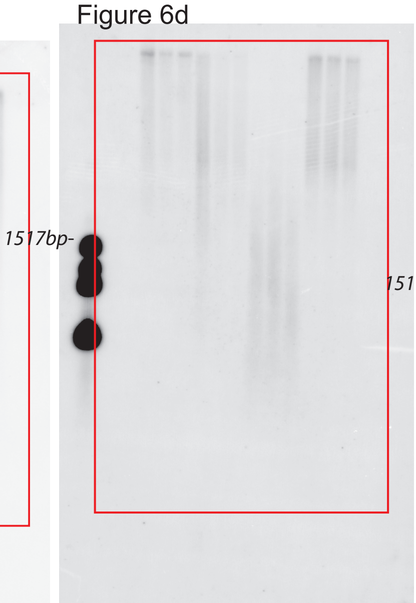

Extended Data Figure 9f

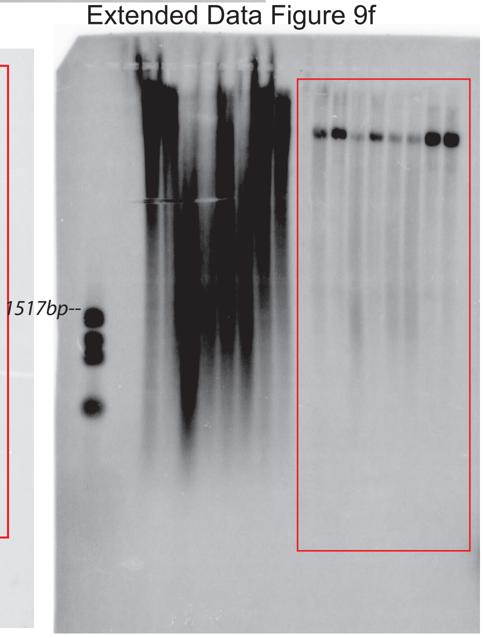

Extended Data Figure 9g

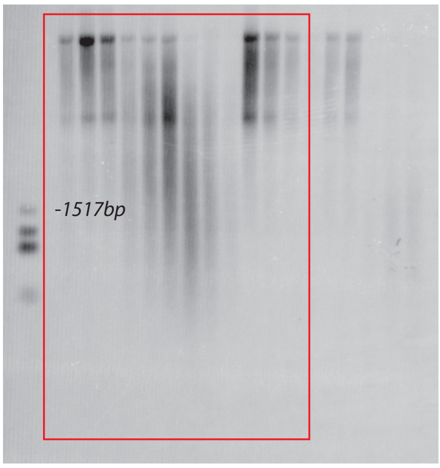

Extended Data Figure 10b

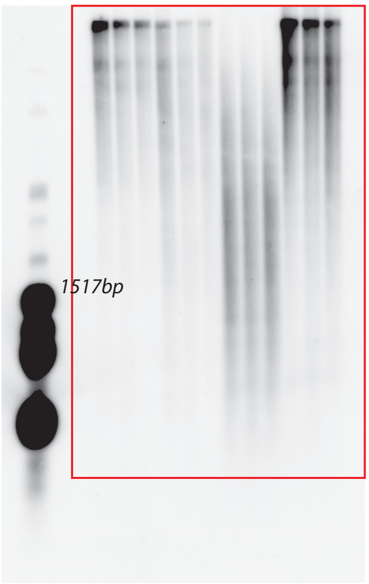

Extended Data Figure 10d

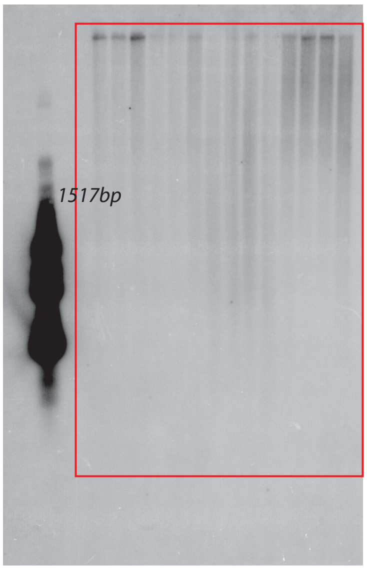

Extended Data Figure 4c

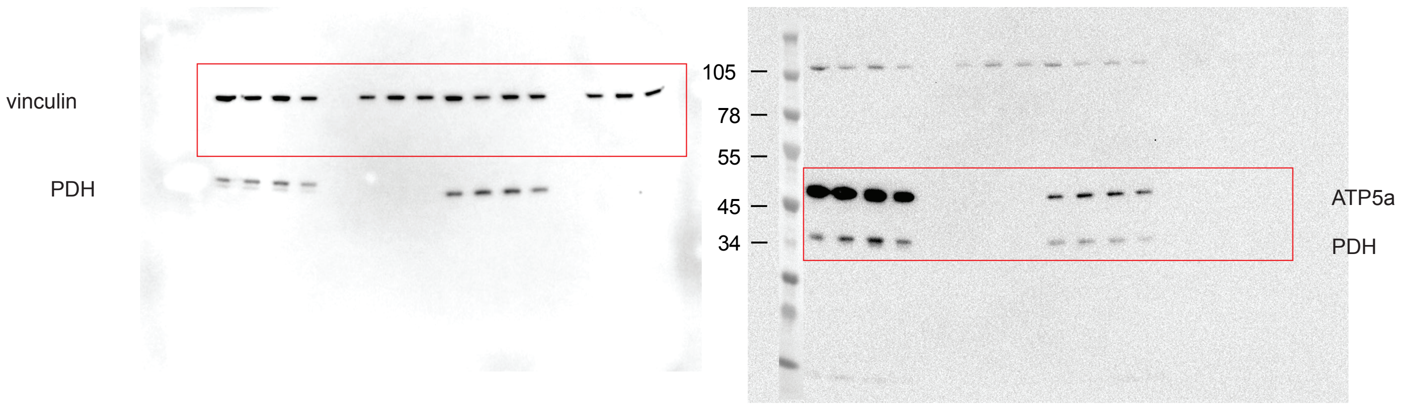

Extended Data Fig. 6c

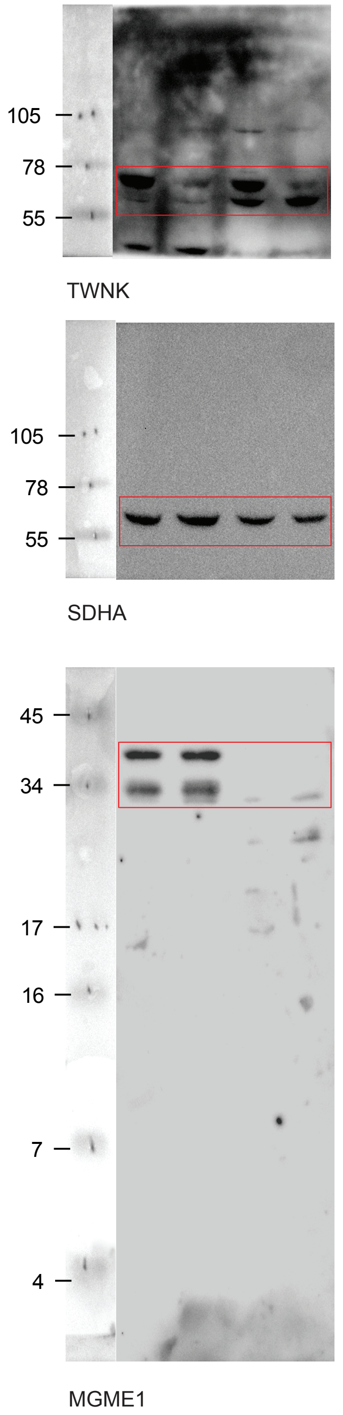

Extended Data Fig. 7b

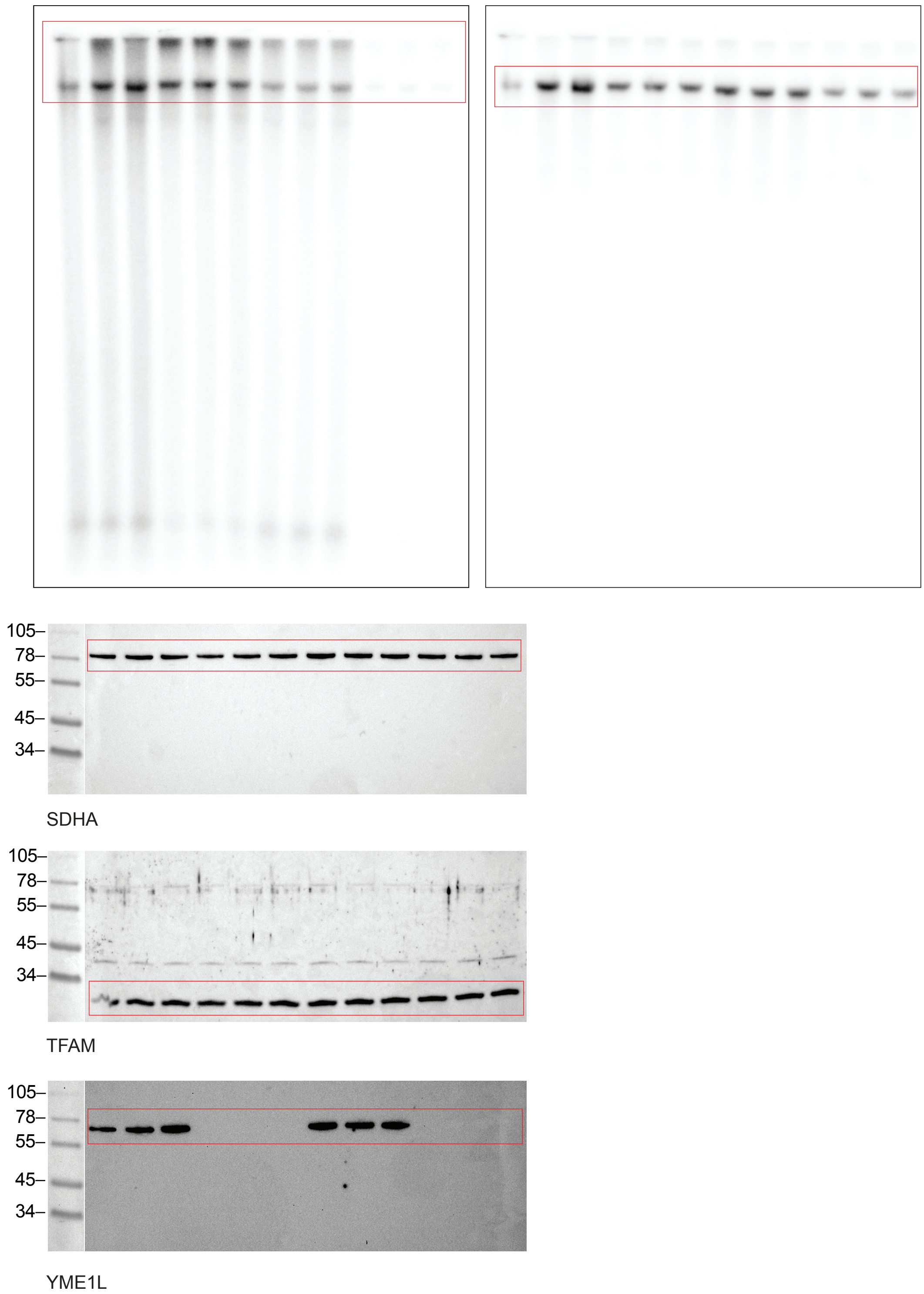

Extended Data Fig. 7c

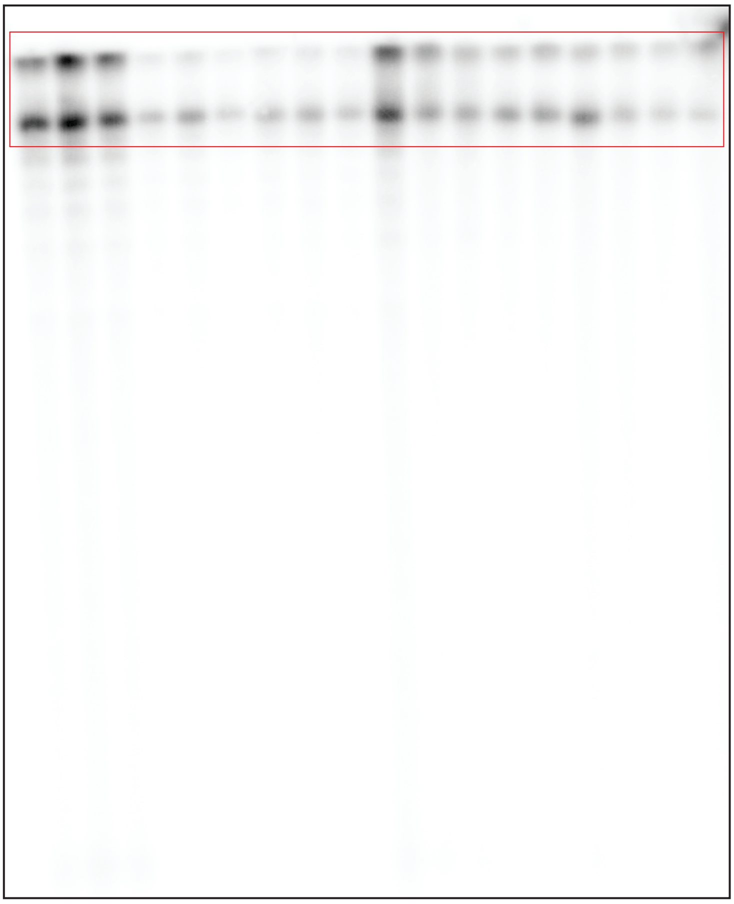

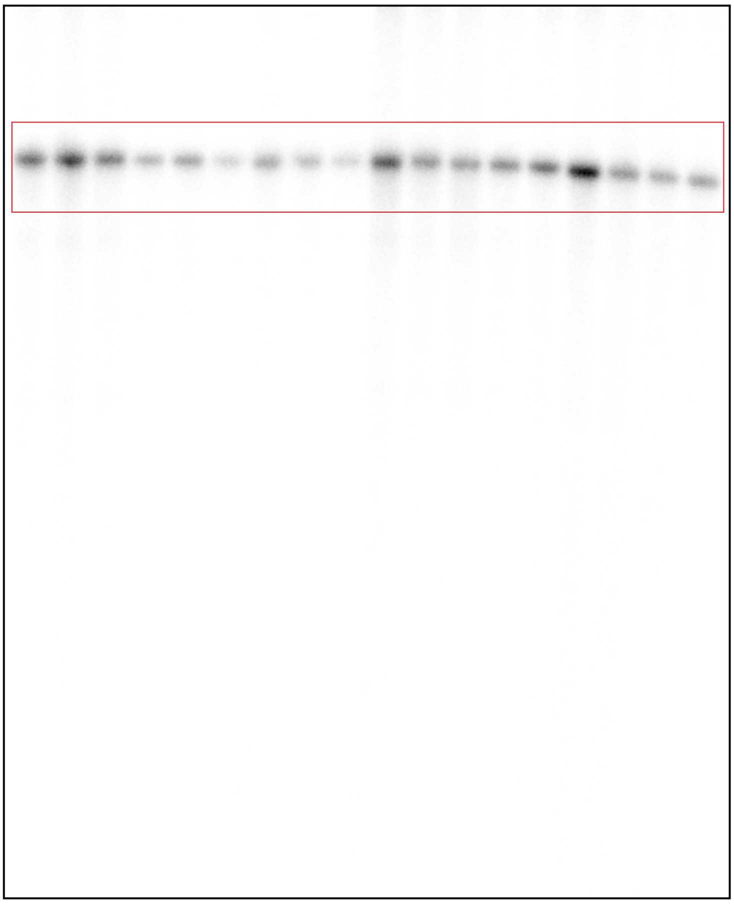

Extended Data Fig. 8c

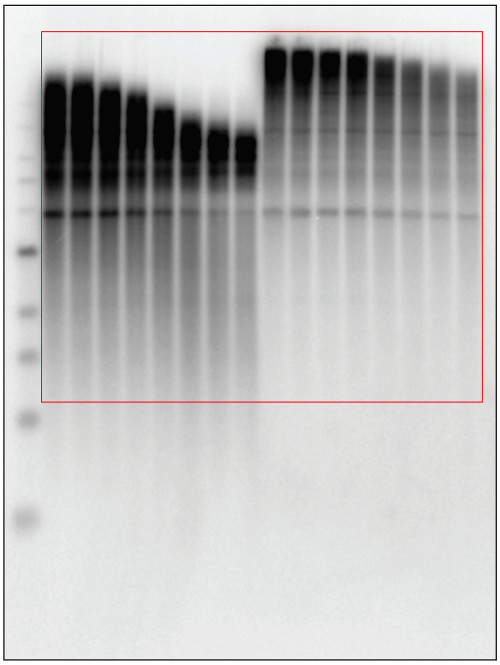

Extended Data Fig. 8d

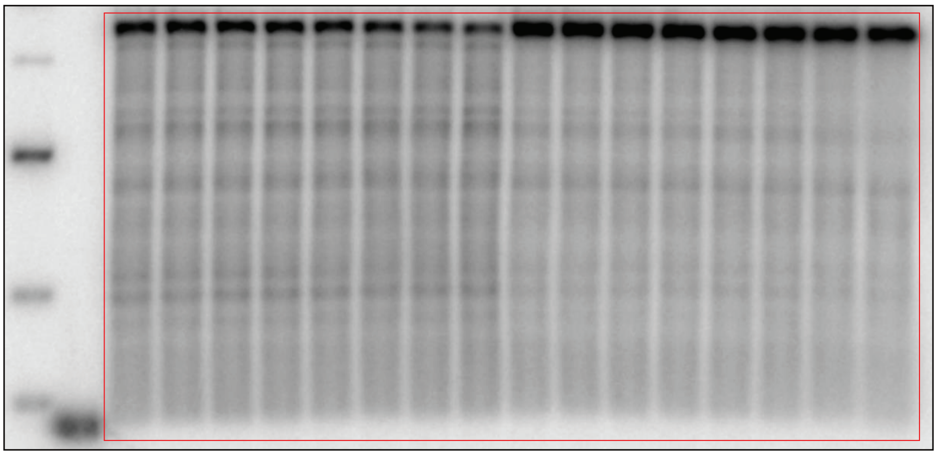

Extended Data Fig. 9c

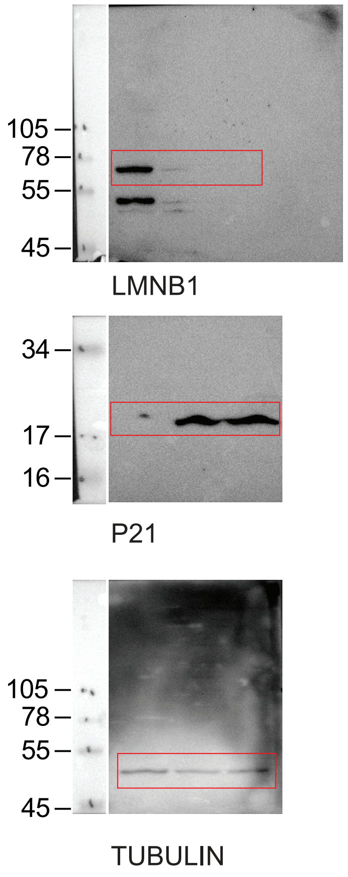

Extended Data Fig. 9i

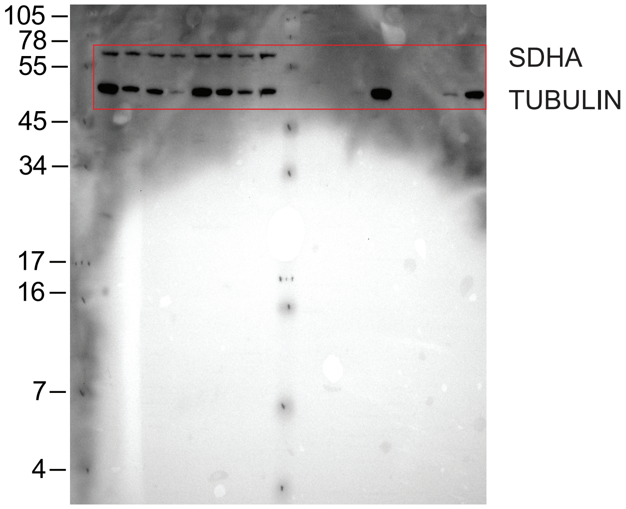

Extended Data Fig. 9d

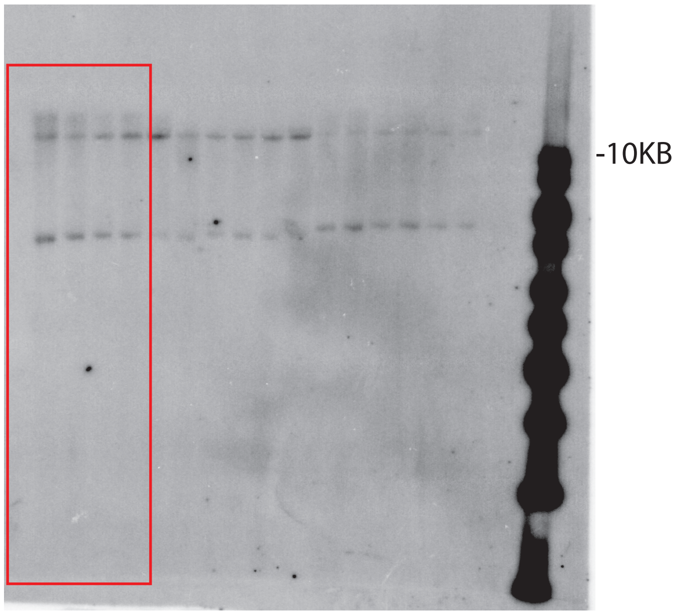

Supplement: Supplementary file 1 — Original scan data. [file 41586_2025_9541_MOESM1_ESM.pdf]
