# Supplementary material for: Ribonucleotide incorporation into mitochondrial DNA drives inflammation
Source: Nature. 2025 Sep 24;647(8090):726–34. doi: 10.1038/s41586-025-09541-7 (PMC12629987; doi:10.1038/s41586-025-09541-7)
Supplement: Supplementary file 2 — Reporting Summary [file 41586_2025_9541_MOESM2_ESM.pdf]

nature portfolio

Corresponding author(s): Thomas Langer

Last updated by author(s): July 25, 2025

Reporting Summary

Nature Portfolio wishes to improve the reproducibility of the work that we publish. This form provides structure for consistency and transparency in reporting. For further information on Nature Portfolio policies, see our Editorial Policies and the Editorial Policy Checklist.

Statistics

For all statistical analyses, confirm that the following items are present in the figure legend, table legend, main text, or Methods section.

n/a	Confirmed	
☐	☒	The exact sample size (*n*) for each experimental group/condition, given as a discrete number and unit of measurement
☐	☒	A statement on whether measurements were taken from distinct samples or whether the same sample was measured repeatedly
☐	☒	The statistical test(s) used AND whether they are one- or two-sided<br>*Only common tests should be described solely by name; describe more complex techniques in the Methods section.*
☒	☐	A description of all covariates tested
☐	☒	A description of any assumptions or corrections, such as tests of normality and adjustment for multiple comparisons
☐	☒	A full description of the statistical parameters including central tendency (e.g. means) or other basic estimates (e.g. regression coefficient) AND variation (e.g. standard deviation) or associated estimates of uncertainty (e.g. confidence intervals)
☐	☒	For null hypothesis testing, the test statistic (e.g. *F*, *t*, *r*) with confidence intervals, effect sizes, degrees of freedom and *P* value noted<br>*Give P values as exact values whenever suitable.*
☒	☐	For Bayesian analysis, information on the choice of priors and Markov chain Monte Carlo settings
☒	☐	For hierarchical and complex designs, identification of the appropriate level for tests and full reporting of outcomes
☒	☐	Estimates of effect sizes (e.g. Cohen's *d*, Pearson's *r*), indicating how they were calculated

Our web collection on statistics for biologists contains articles on many of the points above.

Software and code

Policy information about availability of computer code

Data collection	Radioactive signals were developed using Typhoon FLA 9500. qPCR were performed using 384 Quant studio 7 flex real time pCR macine (Thermo fisher). Nanostringprofiling was performed using nCOUNTER SPRINT profiler. Fluorescence images were acquired using confocal TSC SP8 DLS and TSC SP8X microscopes. dd PCR was performed using digital droplet PCR (Biorad) and dPCR using Qiagen QiaCutie .
Data analysis	Fiji software 4;InstantClue 2; GraphPad Prism 10;Multi Gauge V3.0, nSolver analysis software, Adobe Photoshop 23.5.0, Adobe Illustrator 26.5

For manuscripts utilizing custom algorithms or software that are central to the research but not yet described in published literature, software must be made available to editors and reviewers. We strongly encourage code deposition in a community repository (e.g. GitHub). See the Nature Portfolio guidelines for submitting code & software for further information.

Data

Policy information about availability of data

All manuscripts must include a data availability statement. This statement should provide the following information, where applicable:

- Accession codes, unique identifiers, or web links for publicly available datasets
- A description of any restrictions on data availability
- For clinical datasets or third party data, please ensure that the statement adheres to our policy

The mass spectrometry proteomics data have been deposited to the ProteomeXchange Consortium via the PRIDE repository with the dataset identifier PXD053639 (Extended Data Fig. 1d). HydEn seq fastq data is uploaded to ENA (accession number PRJEB87869). The Illumina sequencing data is data freely available in the SRA

database under accession number: PRJNA1251856. The metabolic raw data used in this publication and associated metadata including tables with m/z values and retention times of the extracted metabolites is deposited on Zenodo and can be accessed under 10.5281/zenodo.15241160.

Research involving human participants, their data, or biological material

Policy information about studies with human participants or human data. See also policy information about sex, gender (identity/presentation), and sexual orientation and race, ethnicity and racism.

Reporting on sex and gender	N/A
Reporting on race, ethnicity, or other socially relevant groupings	N/A
Population characteristics	N/A
Recruitment	N/A
Ethics oversight	N/A

Note that full information on the approval of the study protocol must also be provided in the manuscript.

Field-specific reporting

Please select the one below that is the best fit for your research. If you are not sure, read the appropriate sections before making your selection.

☒ Life sciences ☐ Behavioural & social sciences ☐ Ecological, evolutionary & environmental sciences

For a reference copy of the document with all sections, see nature.com/documents/nr-reporting-summary-flat.pdf

Life sciences study design

All studies must disclose on these points even when the disclosure is negative.

Sample size	No sample size was calculated. Based on previous experience and published data for similar experiments sample size was determined. PMID: 3390377, PMID:31695197
Data exclusions	Due to a genotyping error, we had to exclude one knock-out (sample #2) from the Mgme1-/- kidney and, conversely, one sample from each of the other sets for Illumina sequencing was excluded..
Replication	Where applicable the experiments were repeated at least three times and representative experiments were shown. For some mouse experiments involving more than three individual animals, the experiment was not replicated. The number of mice used for the experiments is indicated in the figure legends. Where representative data are shown, the experimental findings were successfully replicated in independent experiments (numbers indicated in respective figure legends).
Randomization	Mice were assigned to experimental groups based on genotypes and age. Cells were allocated by genotype and treatment.
Blinding	Analysis were not blinded because experiments were performed and analysed by the same researchers.

Reporting for specific materials, systems and methods

We require information from authors about some types of materials, experimental systems and methods used in many studies. Here, indicate whether each material, system or method listed is relevant to your study. If you are not sure if a list item applies to your research, read the appropriate section before selecting a response.

Materials & experimental systems

n/a	Involved in the study	
☐	☒	Antibodies
☐	☒	Eukaryotic cell lines
☒	☐	Palaeontology and archaeology
☐	☒	Animals and other organisms
☒	☐	Clinical data
☒	☐	Dual use research of concern
☒	☐	Plants

Methods

n/a	Involved in the study	
☒	☐	ChIP-seq
☒	☐	Flow cytometry
☒	☐	MRI-based neuroimaging

Antibodies

Antibodies used	The information regarding the antibodies are provided in Supplementary table 5.
Validation	Antibodies were used according to the manufacturer's recommendation and/or previous published work. PMID: 33903774 PMID: 31695197

Eukaryotic cell lines

Policy information about cell lines and Sex and Gender in Research

Cell line source(s)	IMR90 ,RPTEC and HeLa cell lines were acquired from ATCC. MEFs were described in PMID: 3390377, PMID: 24616225 and PMID: 29572490
Authentication	None of the cell lines was authenticated.
Mycoplasma contamination	All cell lines were regulary tested and were negative for mycoplasma contamination
Commonly misidentified lines (See ICLAC register)	No commonly misidentified cell lines were used.

Animals and other research organisms

Policy information about studies involving animals; ARRIVE guidelines recommended for reporting animal research, and Sex and Gender in Research

Laboratory animals	Mus musculus C57BL/6N mouse strain and mice of mixed C57BL/6N x C57BL/6J background were used. C57BL/6N mice of 2w, 55w,80-86w and mixed C57BL/6N x C57BL/6J of 10, 35,55,70w of age were used for experiemnts as reported in the figure legends/ and or method section.
Wild animals	The study did not use wild animals
Reporting on sex	Animals of both sexes were used in the study
Field-collected samples	The study did not used samples collected from the field.
Ethics oversight	The study was approved by the Landesamt für Natur, Umwelt und Verbraucherschutz Nordrhein–Westfalen (reference numbers 81.02.04.2020.A082, 81-02.04.2022.A453) and performed in accordance with the recommendations and guidelines of the Federation of European Laboratory Animal Science Associations (FELASA).

Note that full information on the approval of the study protocol must also be provided in the manuscript.

Plants

Seed stocks	*Report on the source of all seed stocks or other plant material used. If applicable, state the seed stock centre and catalogue number. If plant specimens were collected from the field, describe the collection location, date and sampling procedures.*
Novel plant genotypes	*Describe the methods by which all novel plant genotypes were produced. This includes those generated by transgenic approaches, gene editing, chemical/radiation-based mutagenesis and hybridization. For transgenic lines, describe the transformation method, the number of independent lines analyzed and the generation upon which experiments were performed. For gene-edited lines, describe the editor used, the endogenous sequence targeted for editing, the targeting guide RNA sequence (if applicable) and how the editor was applied.*
Authentication	*Describe any authentication procedures for each seed stock used or novel genotype generated. Describe any experiments used to assess the effect of a mutation and, where applicable, how potential secondary effects (e.g. second site T-DNA insertions, mosiacism, off-target gene editing) were examined.*
