# Supplementary material for: Ribonucleotide incorporation into mitochondrial DNA drives inflammation
Source: Nature. 2025 Sep 24;647(8090):726–34. doi: 10.1038/s41586-025-09541-7 (PMC12629987; doi:10.1038/s41586-025-09541-7)
Supplement: Supplementary file 3 — Supplementary Tables 1–6. [file 41586_2025_9541_MOESM3_ESM.pdf]

Table 1 siRNA / esiRNA used in this study

esiRNA Egfp	EHUEGFP Sigma/Merck	5' – GTGAGCAAGGGCGAGGAGCTGTTCACCGGGGTGGTGCCCATCCTGGTCGAGCTGGACGGCGACGTAAACGGCCACAAGTTCAGCGTGTCCGGCGAGGGCGAGGGCGATGCCACCTACGGCAAGCTGACCCTGAAGTTCATCTGCACCACCGGCAAGCTGCCCGTGCCCTGGCCCACCCTCGTGACCACCCTGACCTACGGCGTGCAGTGCTTCAGCCGCTACCCCGACCACATGAAGCAGCACGACTTCTTCAAGTCCGCCATGCCCGAAGGCTACGTCCAGGAGCGCACCATCTTCTTCAAGGACGACGGCAACTACAAGACCCGCGCCGAGGTGAAGTTCGAGGGCGACACCCTGGTGAACCGCATCGAGCTGAAGGGCATCGACTTCAAGGAGGACGGCAACATCCTGGGGCACAAGCTGGAGTACAACTACAACAGCCACAACGTCTATATCATGGCCGACAAGCAGAAGAACGGCATCAAGGTGAACTTCAAGATCCGCCACAACATCGAGGACGGCAGCGTGCAGCTCGCCGACCACTACCAGCAGAACACCCCCATCGGCGACGGCCCCGTGCTGCTGCCCGACAACCACTACCTGAGCACCCAGTCCGCCCTGAGCAAAGACCCCAACGAGAAGCGCGATCACATGGTCCTGCTGGAGTTCGTGACCGCCGCCGGGATCACTCTCGGCATGGACGAGCTGTA – 3'
siRNA Mgme1 #1	MSS232730 ThermoFischer Scientific	5'-CAAAGGAACTCGATACCCAG-3'
siRNA Mgme1 #2	SASI_Mm01_00073954 Sigma/Merck	5' – CTGCAGGCTGCTTAGCAGGT – 3'
esiRNA PolgA	EMU063481 Sigma/Merck	5'-CGAATGATCCGAAGAGAAGCTTCAAGGAAGTCACGATGGAAGAAGTGGGAGGTAGCCTCTGAACGAGCATGGACAGGGGGCACAGAGTCAGAAATGTTTAATAAGCTGGAGAGTATTGCCATGTCTGATACACCACGTACCCCAGTACTGGGCTGCTGCATCAGCAGAGCCTTGGAGCCCTCAGTTGTCCAGGGAGAGTTTATAACCAGTCGTGTGAACTGGGTGGTACAGAGCTCTGCTGTAGACTACTTACATCTCATGCTTGTGGCCATGAAGTGGCTGTTTGAGGAATTTGCCATTGATGGGCGCTTCTGCATCAGCATCCACGACGAGGTTCGCTACCTGGTGCGTGAGGAGGACCGCTACCGTGCCGCCCTGGCACTGCAGATCACCAATCTCCTGACCAGGT - 3'
esiRNA Twnk	EMU034181 Sigma/Merck	5´-CACGGACAACAGAGCATCAGGTCCGTCATAGACACAATGCAACATGCTGTCTACGTCTATGACGTCTGTCATGTAGTCATTGACAACTTGCAGTTCATGATGGGTCACGAGCAGCTCTCCTCTGACAGGATTGCAGCTCAAGACTACATTGTTGGAGCCTTTCGGAAGTTTGCTACAGACAACAGTTGCCACGTGACTCTGGTCATTCACCCTCGGAAAGAAGAC

		GACGACAAGGAACTGCAGACAGCATCCATTTTCGGCTCAGCCAAAGCAAGCCAAGAAGCAGACAATGTTCTGATCTTACAGGATAGGAAACTGGTGACTGGGCCTGGGAAACGGTATCTGCAGGTGTCCAAGAATCGCTTTGATGGAGACGTAGGTGTCTTCCCACTGGAGTTCAACAAGAATTCCCTTACCTTCTCCATCCCACCCAAGAGCAAAGCCCGACTCAAGAAGATCAAGGACGACAATGGACTAGTGGCC-3´
esiRNA Ssbp1	EMU091191 Sigma/Merck	5’ – TGTCCGGAAAAGCCTAAAGATTAGGTTGTAAGAAAAACAGAAGCCATGTTTCGAAGACCTGTGTTACAGGTATTTCGTCAGTTTGTAAGACATGAGTCTGAAGTAGCCAGCAGTTTGGTTCTTGAACGATCTCTGAATCGTGTTCAGTTACTTGGACGAGTAGGTCAGGACCCTGTCATGAGACAGGTGGAAGGAAAAAACCCAGTCACAATATTTTCTCTAGCAACAAATGAGATGTGGCGATCAGGGGATAGTGAAGTATACCAAATGGGTGACGTTAGTCAGAAGACGACGTGGCACAGAATATCAGTGTTTCGACCAGGCCTCAGAGATGTGGCATATCAGTATGTGAAAAAGGGGGCTCGTATATTTGTGGAAGGGAAAGTGGACTATGGCGAGTACATGGA – 3’
esiRNA Rnaseh1	EMU023091 Sigma/Merck	5’ – CAAGGCTCAGAACATCAGCAAGCTGGTTCTGTACACAGACAGCATGTTCACCATCAATGGGATAACTAACTGGGTTCAGGGCTGGAAGAAGAATGGCTGGAGAACAAGTACAGGGAAAGATGTGATCAACAAGGAGGACTTCATGGAGCTGGACGAGCTCACTCAGGGCATGGACATCCAGTGGATGCACATTCCTGGTCACTCAGGATTTGTGGGCAATGAAGAGGCCGACAGACTGGCACGGGAAGGAGCGAAGCAGTCTGAGGACTGAGCAGAGGAGCTGCTGCCGACTCAGACCCAAGGCAGAGGCTGTTTCTTTACCCTGCCGTGCACTGAGCTGTGCATGGAGGACACAGTGTAACCTGTGGCACAGCTTGGTGAGACGTGCTTACATAATGGAAAGGAAGTGTTAAATCCTGCTTTTGTGAGATTTCAGGGTCTGTCCCAA – 3’
esiRNA Polrmt	EMU033791 Sigma/Merck	5’ – GGAAGTTGACCAGCAGAAGCAAGCCCTCACACAGGAGTTCTGGACCCTTCACAAGGAGCCCAAGATCTGGAACAAGAAGCTGGCTGGCTACCTGCAGCCAAGCAAGAAGGGAACACCCACGAACTCAGAGGAAAAGCAGCTGGCCCAGGCCCTTCAGGCTGCTCTGGGGAGGCTCAGCTCCCGTGAGGCAGAGGCCCTGGCCAGGAAGAAAGCCAAGGCGGTGGAGGCGCAGATCCTGGTCCTCCAGCAGAAGTTCCTGGCTTTCTTTGAGTGCTGCGTCTGCACTGGCCAAGTGCCCCTCGCTCACCACGTGCTGGTCACTCACCATAACAACGGAGACAGACAGCAGGTGCTCACACTGCACATGTACAACACCGTGATGCTTGGCTGGGCCCGCAAGGGCTCCTTCAGAGAGCTGGTCTATGTGTTCCTCATGCTGAAGGATGCTGGCCTCTCCCCAGACCTGTGCTCCTATGCAGCT – 3’
siRNA Samhd1	SASI_Mm01_00193980 Sigma/Merck	5’-AAATCTGTATGACATGTTCCA–3’
esiRNA cGAS	EMU074131	5’ – GGAACCGGACAAGCTAAAGAAGGTGCTGGACAAATTGAGATTGAAACGCAAAGATATCTCGGAGGCGGCCGAGACGGTGAATAAAGTT

	Sigma/Merck	GTGGAACGCCTGCTGCGCAGAATGCAGAAACGGGAGTCGGAGTTCAAAGGTGTGGAGCAGCTGAACACTGGCAGCTACTATGAACATGTGAAGATTTCTGCTCCTAATGAATTTGATGTTATGTTTAAACTGGAAGTCCCCAGGATTGAGCTACAAGAATATTATGAAACAGGTGCTTTTCTATCTTGTGAAATTCAAAAGAATTCCACGAGGAAATCCGCTGAGTCATTTCTTAGAAGGGGAAGTATTATCAGCTACCAAGATGCTGTCAAAGTTTAGGAAAATCATTAAAGAAGAAGTTAAAGAAATCAAAGATATAGATGTCAGTGTGGAGAAGGAAAAACCAGGAAGCC – 3’
esiRNA Sting	EMU074691 Sigma/Merck	5’ – ACCTCCTCCCTCCGTACTGTCCCAAGAGCCAAGACTCCTCATCAGTGGTATGGATCAGCCTCTCCCACTCCGCACTGACCTCATCTGAGGCATGGGACAGCCTTGTCTGGGCTCTAGTGATCCTTTAGCCTCCTGACTGAGCCTTCCTTCAATGGTTGGGGGCCTCAGAGACTTCACATCTCCAGATGAGTCCCACATTCCTGGGCAAGCCATTTATTTCACCTCTCTGAGCCTCAACCAACCCTACTATGAAAGGAGGTCATAATGCGTTCCCTGCCCAGCCAAAGGATTTTATATATGTAGAAGTTGGTGTCAATGCCTGGTAAACTTGAGAGAAAGGCCAAGTACTTCCCGTGGATGCTGCAGACATTCCCTGCTCTCTGTTGACCTGTGTGGATGGTACCAGCAGACTTCCAACCCTCCAGCTTCTGGTCACGTGTGT – 3’
siRNA Mavs	SASI_Mm01_00042222 Sigma/Merck	5’ – TCAGCCCTCCAGAGAGCATC – 3’
siRNA Rig1	SASI_Mm01_00145086 Sigma/Merck	5’ – TGTGGATTGTTGATAAAGGT – 3’
siRNA Mda5	SASI_Mm02_00332785 Sigma/Merck	5’ – TGGTGTTGGAGCAGCCAAAA – 3’
siRNA Slc25a33 #1	SASI_Mm02_00332248 Sigma/Merck	5’ – CGTGCCTAATAGCAATACTG – 3’
siRNA Slc25a33 #2	MSS230288 ThermoFischer Scientific	5’ - TTTGGTTGGAGTTGCACCAT – 3’

esiRNA Polg2	EMU047361-Sigma/Merck	GGGACAGTGCCTTCAGGTTAGTCTCTCCAGAAAGTATACGCGAAATCTTGCAAGACAGAGAGCCGAGTAAGGAACAGCTAGTGGCATTTCTTGAGAACTTATTAAAAACTTCTGGGAAACTACGGGCGACTCTTCTTCACGGTGCCTTGGAGCACTATGTTAATTGCCTGGATCTGGTAAACAGAAAGCTACCTTTCGGTCTTGCTCAGATTGGAGTCTGTTTCCATCCTGTTTCGAACAGCAATCAGACACCCAGCAGTGTTACAAGAGTTGGTGAAAAGACGGAAGCTTCCTTGGTATGGTTTACTCCCACAAGGACTTCGAGCCAGTGGCTTGATTTCTGGTTGCGTCATCGGCTTCTGTGGTGGAGAAAGTTTGCTATGAGTCCCTCTAACTTCAGCAGCGCTGA
siRNA mtSSBP1	SASI_Mm02_00333734 Sigma/Merck	

Table 2: A) SYBR Green primers (mouse) used for qPCR

Hprt	fwd	5’- TCCTCCTCAGACCGCTTTT -3’
	rev	5’- CATAACCTGGTTCATCATCGC -3’
Isg15	fwd	5’- CTAGAGCTAGAGCCTGCAG -3’
	rev	5’- AGTTAGTCACGGACACCAG -3’
Ifi44	fwd	5’- CTGATTACAAAAGAAGACATGACAGAC -3’
	rev	5’- AGGCAAAACCAAAGACTCCA -3’
Usp18	fwd	5’- GAGAGGACCATGAAGAGGA -3’
	rev	5’- TAAACCAACCAGACCATGAG -3’
Mx-1	fwd	5’- GACCATAGGGGTCTTGACCAA -3’
	rev	5’- AGACTTGCTCTTTCTGAAAAGCC -3’
Ifit3	fwd	5’- TTCCCAGCAGCACAGAAAC -3’
	rev	5’- AAATTCCAGGTGAAATGGCA -3’
Zbp1	fwd	5’- TGTTGACTTGAGCACAGGAG -3’
	rev	5’- TTCAGGCGGTAAAGGACTTG -3’
cGAS	fwd	5’- GTGAGGACCAATCTAAGACGAG -3’

	rev	5’- AGCATGTTTTCTCTATCCCGTG -3’
Sting	fwd	5’- CTCATTGTCTACCAAGAACC -3’
	rev	5’- TAACCTCCTCCTTTTCTTCC -3’
Yme1l	fwd	5’-GCCAGATGTGAAGGGTCGAACT-3´
	rev	5’-ACTCTGCTCCAGAGAACCCAAC-3´
Samhd1	fwd	5’- AAGTGGAGTACAAGGTCAAG -3’
	rev	5’- GATTTCCAACCTCCTTTTCTC -3’
Mgme1	fwd	5’-GAGGTGGAAAGAGCGGATGGTT -3`
	rev	5’-GGTGACAGTATGCTTTCCAAGGC -3`
Slc25a33	fwd	5‘ – ATGTGCCTCCTGCATCGCTTAC – 3‘
	rev	5‘ – GCAAACAGTCCTCGGTAGAAGG – 3‘
Twnk	fwd	5‘ – GTCTGCTGAAGGGACATCGGAA – 3‘
	rev	5‘ – GGCTAGTCTCACGTTGCTGATC – 3‘
Rig1	fwd	5‘ – TTGAAAGACTTGGGTACAAC – 3‘
	rev	5‘ – TCATCAGAGTGAAGACAGAC – 3‘

Mda5	fwd	5‘ – ACAGTGAAAGAGAATCTTGG – 3‘
	rev	5‘ – CTGCTCATAATGTTGGGTTC – 3‘
Mavs	fwd	5‘ – CAGTAGAGAATTCAGAGCAAC – 3‘
	rev	5‘ – TTAGGAGAGGGTATCAAAGAG – 3‘
Polga	fwd	5‘ – AGGAAGCACTGCCTTGAACAGG – 3‘
	rev	5‘ – CTCAGCCTCTACGTCTAAGCAG – 3‘
Ssbp1	fwd	5‘ – CAGAAGACGACGTGGCACAGAA – 3‘
	rev	5‘ – TGTTGCTTGCCGCCTCACATTG – 3‘
Rnaseh1	fwd	5‘ – GCTGTAGTGAGCAAGGACGCAT – 3‘
	rev	5‘ – CAGTAAACGCCAATTCCTGCTCG – 3‘
ActB	fwd	5’ – CATTGCTGACAGGATGCAGAAGG – 3’
	rev	5’ – TGCTGGAAGGTGGACAGTGAGG – 3’
Dloop1	fwd	5’ – AATCTACCATCCTCCGTGAAACC – 3’
	rev	5’ – TCAGTTTAGCTACCCCCAAGTTTAA – 3’
CytB	fwd	5’ – GCTTTCCACTTCATCTTACCATTTA – 3’

	rev	5’ – TGTTGGGTTGTTTGATCCTG – 3’
Non-NUMT	fwd	5’ – CTAGAAACCCCGAAACCAAA – 3’
	rev	5’ – CCAGCTATCACCAAGCTCGT – 3’

Table 2: B) SYBR Green primers (human) used for qPCR -

Hprt	fwd	5’- TGACACTGGCAAAACAATGCA -3’
	rev	5’- GGTCCTTTTCACCAGCAAGCT -3’
Cxcl1	fwd	5’- AGCTTGCCTCAATCCTGCATCC -3’
	rev	5’- TCCTTCAGGAACAGCCACCAGT -3’
Cxcl2	fwd	5’- GGCAGAAAGCTTGTCTCAACCC -3’
	rev	5’- CTCCTTCAGGAACAGCCACCAA -3’
Il1b	fwd	5’- CCACAGACCTTCCAGGAGAATG -3’
	rev	5’- GTGCAGTTCAGTGATCGTACAGG -3’
Il8	fwd	5’- GAGAGTGATTGAGAGTGGACCAC -3’
	rev	5’- CACAACCCTCTGCACCCAGTTT -3’
Icam1	fwd	5’- AGCGGCTGACGTGTGCAGTAAT -3’
	rev	5’- TCTGAGACCTCTGGCTTCGTCA -3’

Lmnb1	fwd	5’- GAGAGCAACATGATGCCCAAGTG -3’
	rev	5’- GTTCTTCCCTGGCACTGTTGAC -3’

Table 3: PCR primers and probes used for Southern blot analysis

Target	Primer Sequence		Probe Sequence
m18S	fwd		TCCCAAGATCCAACTACGAGCTTTTTAACT<br>GCAGCAACTTTAATATACGCTATTGGAGCT<br>GGAATTACCGCGGCTGCTGGCACCAGAC<br>TTGCCCTCCAATGGATCCTCGTTAAAGGA<br>TTTAAAGTGGACTCATTCCAATTACAGGGC<br>CTCGAAAGAGTCCTGTATTGTTATTTTTCG<br>TCACTACCTCCCCGGGTCGGGAGTGGGT<br>AATTTGCGCGCCTGCTGCCTTCCTTGGAT<br>GTGGTAGCCGTTTCTCAGGCTCCCTCTCC<br>GGAATCGAACCCTGATTCCCCGTCACCCG<br>TGGTCACCATGGTAGGCACGGCGACTAC<br>CATCGAAAGTTGATAGGGCAGACGTTCGA<br>AGGGTCGTCGCCGCCACGGGGGGCGTG<br>CGATCGGCCCGAGGTTATCTAGAGTCACC<br>AAAGCCGCCGGCGCCCGACCCCCGGCC<br>GGAGCCGGGAGGGAGCTCACCGGGTTG<br>GTTTTGATCTGATAAATGCACGCATCCCCC<br>CCCGGGAAGGGGGGTCAGCGCCCGTCG<br>GCATGTATTAGCTCTAGAATTACCACAGTT<br>ATCCAAGTAGGAGAGGAGCGAGCGACCA<br>AAGGA<br>ACCATAACTGATTTAATGAGCCATTCGCAG<br>TTTCACTGTACCGGCCGTG
	rev		
h18S	fwd	5´- GTTGGTGGAGCGATTTGTCT - 3´	
	rev	5´- GGCCTCACTAAACCATCCAA - 3´	
mCOX 1	fwd		TGAATATGTGGTGGGCTCATACAATAAAG<br>CCTAGAAAGCCAATAGACATTATTGCTCA<br>TACTATTCCTATATAGCCGAAAGGTTCTTT<br>TTTTCCGGAGTAGTAAGTAACTACATGTG<br>AAATAATTCCAAATCCTGGGAGGATAAGA<br>ATATAAACTTCTGGGTGCCCAAAGAATCA<br>GAACAGATGCTGGTAGAGAATTGGGTCC<br>CCTCCTCCAGCGGGATCAAAGAAAGTTGT<br>GTTTAGGTTGCGGTCTGTTAGTAGTATAG

	rev		TAATGCCTGCGGCTAGCACTGGTAGTGAT<br>AATAGGAGCAGTACGGCTGTAATAAGTAC<br>GGATCAGACAAATAGTGGAGTTTGATACT<br>GTGTTATGGCTGGGGGTTTCATGTTGATA<br>ATAGTGGTAATAAAATTAATTGCACCTAAA<br>ATAGATGACACTCCAGCTAAATGAAGGGA<br>GAAAATTGTTAGGTCTACTGATGCTCCTG<br>CATGGGCTAGATTTCCGGCTAGAGGTGG<br>GTAGACTGTTCATCCTGTTCCTGCTCCTG<br>CTTCTACTATTGATGATGCTAGGAGAAGG<br>AGAAATGATGGTGGTAGGAGTCAAAAACT<br>TATATTATTTATTCGTGGGAATGCTATATC<br>TGGGGCTCCGATTATTAGTGGGACAAGTC<br>AGTTTCCAAAGCCTCCAATTATTATTGGTA<br>TTACTATGAAGAAAATTATAACAAAAGCAT<br>GGGCAGTTACGATAACATTGTAAATTTGG<br>TCATCTCCTAAAAGTGCACCTGGTTGACC<br>TAATTCTGCTCGAATTAAAATACTTAGTGC<br>AGTACCCACTATTCCCGCTCAGGCTCCGA<br>ATAGTAGATAGA
hCOX 1	fwd		ATGAGCTGGAGTCCTAGGCACAGCTCTA<br>AGCCTCCTTATTCGAGCCGAGCTGGGC<br>CAGCCAGGCAACCTTCTAGGTAACGAC<br>CACATCTACAACGTTATCGTCACAGCCC<br>ATGCATTTGTAATAATCTTCTTCATAGTAA<br>TACCCATCATAATCGGAGGCTTTGGCAA<br>CTGACTAGTTCCCCTAATAATCGGTGCC<br>CCCGATATGGCGTTTCCCCGCATAAACA<br>ACATAAGCTTCTGACTCTTACCTCCCTCT<br>CTCCTACTCCTGCTCGCATCTGCTATAG<br>TGGAGGCCGGAGCAGGAACAGGTTGAA<br>CAGTCTACCCTCCCTTAGCAGGGAACTA<br>CTCCCACCCTGGAGCCTCCGTAGACCT<br>AACCATCTTCTCCTTACACCTAGCAGGT<br>GTCTCCTCTATCTTAGGGGCCATCAATTT<br>CATCACAACAATTATCAATATAAAACCCC<br>CTGCCATAACCCAATACCAAACGCCCCT<br>CT
	rev		

Table 4: PCR Primers/probes set used for dPCR and ddPCR

Target		Primer Sequence	Probe Sequence	Fluorophore
mActB	fwd	5´-CCGTTCCGAAAGTTGCCTTT-3´	5´-GCCACTGTCGAGTCGCGTCCACCCG-3´	Cy5
	rev	5´-CTGCAAAGAAGCTGTGCTCG-3´		
mCytB	fwd	5´-GCTTTCCACTTCATCTTACCATTTA-3´	5´-TAGCAATCGTTCACCTCCTCTTCCTCC-3´	ROX
	rev	5´-TGTTGGGTTGTTTGATCCTG-3´		
mDloop	fwd	5´-AATCTACCATCCTCCGTGAAACC-3´	5´-ACCAATGCCCCTCTTCTCGCTCC-3´	FAM
	rev	5´-TCAGTTTAGCTACCCCCAAGTTTAA-3´		
mNd1	fwd	5´-GAGCCTCAAACTCCAAATACTCACT-3´	5´-CCGTAGCCCAAACAAT-3´	TAMRA
	rev	5´-GAACTGATAAAAGGATAATAGCTATGGTTACTTCA-3´		

mCox3	fwd	5´-CCTCGTACCAACACACATGATCTAGG-3´	5´-ACCTCCAACAGGAATTTCA-3´	HEX
	rev	5´-AGTGGGACTTCTAGAGGGTTAAGTG-3´		
mNd4	fwd	5´-GAAGCAACCTTAATCCCAACAC-3´	5´-ATGAGGGAACCAAACTGAACGCCT-3´	Cy5
	rev	5´-AGCAGTGGAATAGAACCGATTAG -3´		
m12S	fwd	5´-CTACCTCACCATCTCTTGCTAAT-3´	5´-ATACCGCCATCTTCAGCAAACCCT -3´	FAM
	rev	5´-TTGGCTACACCTTGACCTAAC-3´		
m16S	fwd	5´-GGACATCCCAATGGTGTAGAA-3´	5´-CCTACGTGATCTGAGTTCAGACCGGA-3´	HEX
	rev	5´-AGATAGAAACCGACCTGGATTG-3´		
hDloop	fwd	5´-CTTGACCACCATCCTCCGTG-3´	5´ACTCTCCTCGCTCCGGGCCCA-3´	HEX
	rev	5´-TGAAGTAGGAACCAGATGTCGG-3´		

Table 5: Primary Antibodies used for immunofluorescence and immunoblotting

Ab Target	Manufacturer	Dilution	Host Organism/ Isoform
mTOMM20	Protein Tech #66777-1-Ig	1:500 (IF)	Mouse/IgG2b
mTOMM20	Abcam #ab232589	1:1000 (IF)	Rabbit/IgG
mTOMM20	Abcam # ab56783	1:500 (IF)	mouse/ IgG1
mPDH	Abcam #ab110333	1:1000 (IF)	mouse/ IgG2a
DNA	Millipore #CBL186	1:750 (IF)	Mouse/ IgM
mcGAS	Millipore #31659	1:500 (IF)	Rabbit/ IgG
mSLC25A33	Origene #TA309042	1:1000 (WB)	Rabbit/IgG
hLMNB1	Abcam #ab16048	1:1000 (WB)	Rabbit/IgG
hP21	Cell Signalling #2947	1:1000 (WB)	Rabbit/IgG
hTUBULIN	Sigma/Merck #T6074	1:1000 (WB)	Mouse/IgG1
mTWINKLE	selfmade	1:1000	Rabbit/IgG
mMGME1	selfmade	1:1000	Rabbit/IgG
mATP5a	Abcam ab14748	1:1000	Mouse/IgG2b
hVinculin	Cell signaling 4650	1:1000	Rabbit/IgG
hYME1l	Proteintech (11510-1-AP)	1:1000	Rabbit/IgG

Ab Target	Manufacturer	Dilution	Host Organism/ Isoform
CD3	Cell signaling 99940	1:100 (IHC)	Rabbit/IgG
B220	BD Pharmingen 550286	1:50 (IHC)	Rat

Compound	Precursor	Fragment	dwell time	cone V	collision energy (V)	Ionization mode	Retention Time (min)
ADP	425,9	158,9	0,063	20	28	[M-H+]-	15,94
ADP	425,9	272,9	0,063	20	28	[M-H+]-	15,94
ADP_15N5	431,1	159	0,063	12	26	[M-H+]-	10,88
ADP_15N5	431,1	333,1	0,063	12	18	[M-H+]-	10,88
AMP	346	96,9	0,096	10	22	[M-H+]-	18,66
AMP	346	133,9	0,096	10	22	[M-H+]-	18,66
AMP_13C10_15N5	361,1	79	0,096	24	18	[M-H+]-	18,66
AMP_13C10_15N5	361,1	97	0,096	24	20	[M-H+]-	18,66
AMP_13C10_15N5	361,1	144	0,096	24	30	[M-H+]-	13,44
ATP	506,1	159	0,068	10	30	[M-H+]-	13,44
ATP	506,1	408	0,068	10	30	[M-H+]-	8,48
ATP_13C10	516,16	158,98	0,068	38	36	[M-H+]-	8,48
ATP_13C10	516,16	418,16	0,068	38	20	[M-H+]-	16,87
CDP	401,9	158,9	0,096	32	25	[M-H+]-	16,87
CDP	401,9	303,9	0,096	32	18	[M-H+]-	18,48
CMP	322	96,9	0,107	20	22	[M-H+]-	18,48
CMP	322	210,9	0,107	20	16	[M-H+]-	16,31
CTP	481,9	384	0,063	20	20	[M-H+]-	16,31
CTP	481,93	158,9	0,063	20	22	[M-H+]-	16,31
dATP	490,1	159	0,068	10	32	[M-H+]-	16,31
dATP	490,1	391,9	0,068	10	25	[M-H+]-	13,65
dCMP	306,16	79,07	0,063	10	22	[M-H+]-	13,65
dCMP	306,16	96,97	0,063	10	20	[M-H+]-	13,65
dCMP	306,16	177,03	0,063	10	18	[M-H+]-	16,3
dCMP	306,16	195,07	0,063	10	18	[M-H+]-	16,3
dCTP	465,9	158,81	0,063	20	30	[M-H+]-	21,27
dCTP	465,9	368,1	0,063	20	20	[M-H+]-	21,27
dGTP	505,98	158,9	0,092	20	34	[M-H+]-	21,27
dGTP	505,98	408	0,092	20	20	[M-H+]-	13,64
dGTP	506,98	426,2	0,092	20	24	[M-H+]-	13,64
dTMP	321,2	79	0,122	4	24	[M-H+]-	19,7
dTMP	321,2	195,1	0,122	4	4	[M-H+]-	19,7
dTTP	480,8	158,9	0,068	20	46	[M-H+]-	20,13
dTTP	480,8	383	0,068	20	20	[M-H+]-	20,13
dUMP	307,16	79,01	0,096	4	28	[M-H+]-	17,27
dUMP	307,16	111,04	0,096	4	20	[M-H+]-	17,27
dUMP	307,16	195,08	0,096	4	12	[M-H+]-	21,8
GDP	441,9	150	0,068	20	30	[M-H+]-	21,8
GDP	441,9	344	0,068	20	16	[M-H+]-	16,95

GMP	362,1	78,9	0,066	30	24	[M-H+]-	16,95
GMP	362,1	210,96	0,066	30	14	[M-H+]-	18,2
GTP	521,9	158,9	0,139	36	32	[M-H+]-	18,2
GTP	521,9	424	0,139	36	32	[M-H+]-	14,79
IMP	346,9	78,9	0,063	20	18	[M-H+]-	14,79
IMP	346,9	96,9	0,063	20	20	[M-H+]-	20,17
UDP	402,9	158,9	0,063	34	26	[M-H+]-	20,17
UDP	402,9	110,9	0,063	34	20	[M-H+]-	15,94
UMP	322,9	96,9	0,079	20	20	[M-H+]-	15,94
UMP	322,9	210,9	0,079	20	12	[M-H+]-	10,88
UTP	482,9	158,8	0,068	20	20	[M-H+]-	10,88
UTP	482,9	385	0,068	20	20	[M-H+]-	10,88
